# The induction of skin tumours in mice by neonatal injection of 9,10-dimethyl-1,2-benzanthracene (DMBA) followed by applications of croton oil to the skin.

**DOI:** 10.1038/bjc.1967.41

**Published:** 1967-06

**Authors:** M. A. Walters, F. J. Roe


### THE INDUCTION OF SKIN TUMOURS IN MICE BY NEONATAL INJECTION OF 9,10-DIMETHYL-1,2-BENZANTHRACENE (DMBA) FOLLOWED BY APPLICATIONS OF CROTON OIL TO THE SKIN

MARGARET A. WALTERS AND F. J. C. ROE

*From the Chester Beatty Research Institute, Institute of Cancer Research: Royal Cancer Hospital, Fulham Road, London, S.W.3*

Received for publication December 12, 1966

CHIECO-BIANCHI *et al.* (1963) reported that Swiss mice receiving urethane as a subcutaneous injection at birth (1 mg./g. body weight) followed by twice weekly applications of croton oil, starting at forty days, developed skin papillomas. The tumour incidence was significantly greater than that in mice given urethane or croton oil only, and that in mice given an equivalent dose (on a body weight basis) of urethane at forty days and similar treatment with croton oil.

Graffi, Scharsach and Heyer (1955) found that doses of 400–1200 $\mu$g. 9,10-dimethyl-1,2-benzanthracene (DMBA) effectively initiated the induction of skin tumours when given orally, intravenously, or intraperitoneally to adult mice. Croton oil, applied to the skin, was the promoter. Small doses of DMBA induce malignant lymphomas, lung adenomas and a wide variety of other tumours when injected into newborn mice (Pietra, Spencer and Shubik, 1959; Pietra, Rappaport and Shubik, 1961; Roe, Rowson and Salaman, 1961; Toth, Rappaport and Shubik, 1962, 1963). The present experiments were designed to see whether DMBA injected, subcutaneously, in low doses during the neonatal period has an initiating effect on the skin.

### MATERIALS AND METHODS

*Chemicals.*—9,10-Dimethyl-1,2-benzanthracene (DMBA) was obtained from Roche Products Ltd. and prepared for injection as a colloidal suspension in 3% aqueous gelatine, using the method of Pietra *et al.* (1959). Gelatine powder was obtained from British Drug Houses. Croton oil, from Messrs. Boots Pure Drug Co., was used as a 0·1% (v/v) solution in acetone.

*Mice.*—Chester Beatty stock mice were used for Experiment 1, and BALB/c (Bittner agent free) mice for Experiment 2. The line of BALB/c was originally obtained from Dr. H. B. Andervont of the National Cancer Institute, Bethesda, Maryland, and has been maintained in this Institute by brother–sister mating since 1952.

Mice were weaned when they were four weeks old and the sexes were separated. Four to six mice were housed together in metal cages. They received a cubed diet (Diet 86, Messrs. Dixon and Sons, Ware, Herts.) and tap water *ad libitum*. As a precaution against ectromelia all mice were vaccinated with sheep lymph when they were about six weeks old.

Before the start of treatment to the skin the hair was removed from the whole back by electric clippers. Croton oil in acetone, acetone only, or, in the case of

Group 4, DMBA in acetone solution, was delivered from calibrated pipettes in such a way that the entire clipped area was covered. The appearance, regression and size of all skin tumours were recorded weekly. Sick mice, which were killed, mice which died and the animals killed at the end of the experiments were all examined thoroughly *post mortem*. A proportion of the skin tumours which arose, and all lesions from other organs which were possibly neoplastic, were taken for microscopic examination.

*Experiment* 1

Twenty-two litters were randomly divided into four groups of forty to fifty mice. The mice in Groups 1 and 2 were injected, subcutaneously, with 45 $\mu$g. DMBA in 0·02 ml. of 3% aqueous gelatine when they were less than twenty-four hours old. Group 3 was similarly treated with 0·02 ml. aqueous gelatine alone. At six weeks, each mouse in Group 4 was painted with 150 $\mu$g. DMBA in 0·3 ml. acetone. Weekly applications of 0·1% croton oil in acetone (Groups 1, 3 and 4) or acetone alone (Group 2) were started during the eighth week. Each mouse received 0·3 ml. per application. Survivors were killed after thirty paintings, i.e. during the thirty-eighth week of the experiment.

RESULTS

Survival from the eighth week to the thirty-sixth week (two weeks before the survivors were killed) and the accumulation of skin tumours are shown in Table I. No skin papillomas were seen in Group 3 (aqueous gelatine and croton oil) and only one was recorded in Group 2 (DMBA and acetone). However, mice of both sexes injected at birth with DMBA and subsequently painted with croton oil (Group 1) developed multiple skin papillomas, although the total number of skin tumours recorded was lower than that in Group 4 and there were fewer tumour-bearing mice. Group 4 was included as a positive control: the mice were treated with DMBA (by application to the skin) as adults, then had the same croton oil treatment as Groups 1 and 3.

The first papillomas appeared during the nineteenth week of the experiment (*i.e.* after eleven applications of croton oil), both in Group 1 and in Group 4. The incidence of skin tumours was calculated from the total of tumour-bearing/mice and the number of survivors at the time of the appearance of the first tumour.

One tumour, from a female in Group 4 killed after thirty-eight weeks, was found on microscopic examination to be a squamous cell carcinoma which was invading the dermis, but had not penetrated the panniculus muscle.

Table II shows the incidence of lung tumours and other neoplasms. The proportion of mice with lung adenomas was similar in Groups 1 and 2, and much higher in these groups than in mice which were not injected with DMBA when newly born. The mean nodule counts were low in Groups 3 and 4 and higher in Group 1 than in Group 2. The difference in lung tumour multiplicity when the neonatal injection of DMBA is followed by treatment with croton oil rather than acetone is significant for females (P = 0·001) but not for males. The mean size of the largest tumour was also greater for the females of Group 1 than for the females of Group 2, but the difference was not significant (P = 0·1). Lung tumours were classified by a method described by Walters (1966). Class 1 includes small well circumscribed adenomas; Class 2, tumours which have invaded adjacent bronchi or bronchioles; Class 3, tumours which have given rise to metastases elsewhere in the lung. Class 3 tumours which were regarded as malignant, and Class 2 tumours, were only seen in mice injected at birth with DMBA.

Pleomorphic sarcomas at the injection site were seen in one mouse in Group 1 and two mice in Group 2. Malignant lymphomas occurred in mice of all groups. Multiple hepatomas were found in males, but not in females. The incidence in Groups 1 and 2 was far greater than in Groups 3 and 4. Females in Groups 1 and 2 developed haemangiomas of the uterus and ovary, and there was one granulosa cell tumour of the ovary in Group 2. One male in Group 1 and one in Group 2 had subcutaneous haemangiomas.

*Experiment* 2

Thirty litters were allotted randomly to six groups. At the start of the experiment there were between forty and fifty mice in each group. The mice in Group 1 were injected subcutaneously with 0·02 ml. of 3% aqueous gelatine, less than twenty-four hours after birth. Group 2 were similarly treated with 5 $\mu$g. DMBA in aqueous gelatine; Group 3 received 15 $\mu$g. DMBA; and Groups 4 and 5 received 45 $\mu$g. DMBA. The mice in Group 6 were injected when they were about eight weeks old (body weights of 18 g. $\pm$ 2 g.). On three successive days they received two subcutaneous injections (one in each flank), of 30 $\mu$g. DMBA. The total dose was 180 $\mu$g. Groups 1, 2, 3, 4 and 6 were painted once each week with 0·25 ml. of 0·1% croton oil in acetone for forty weeks. The treatment was started during the eighth week in Groups 1–4 and during the ninth in Group 6. The control mice in Group 5 received forty weekly applications of 0·25 ml. acetone. The survivors in Groups 1–6 were killed about one week after the end of the treatment with croton oil or acetone.

Group 7 was included as a positive control group to check the susceptibility of BALB/c mice to skin carcinogenesis by DMBA and croton oil. Fifteen twelve-week-old females received one application to the skin of 150 $\mu$g. of DMBA in 0·2 ml. acetone. Weekly treatments of croton oil started three weeks later. The mice received twenty-five applications of 0·25 ml., and were killed at the end of that time.

### RESULTS

Tumour incidence in BALB/c mice after treatment with DMBA and croton oil is shown in Table III. Only three of the fifteen mice in Group 7, painted with 150 $\mu$g. DMBA and croton oil for twenty-five weeks, developed skin papillomas. Each mouse had only one tumour and the earliest appeared after sixteen applications of croton oil. Very few skin tumours arose in Groups 2, 3, 4 and 5. Only one mouse, a female in Group 4, had more than one papilloma: four tumours were recorded after twenty applications of croton oil. Papillomas appeared in one male in Group 4 and one female in Group 5 before the croton oil treatment began.

Twenty-two out of twenty-seven females and ten out of ten males in Group 6 developed pleomorphic or spindle cell sarcomas in one or both flanks. (A dose of 90 $\mu$g. DMBA was deposited at each injection site). Six of the tumours were removed but recurred after three or four weeks. One spindle cell sarcoma was removed successfully. Most of the tumours had invaded the skin or the muscles of the body wall.

One hundred per cent of the survivors in Groups 2, 3, 4, 5 and female survivors in Group 6 developed lung adenomas. The mean nodule count increased between Groups 2 and 3 and Groups 3 and 4, with the increasing dose of neonatally injected DMBA. Females developed more lung tumours than males, but the difference was not significant except in Group 2 where $P < 0{\cdot}05$. There were too few survivors in Groups 4 and 5 for comparison. Tumour multiplicity in mice of Group 4 injected with 45 $\mu$g. DMBA and painted with croton oil was almost the same as that in Group 5 mice, which received the same dose of DMBA, but acetone instead of croton oil. Class 3 and Class 2 lung tumours arose only in mice which were injected with DMBA at birth.

### DISCUSSION

Chester Beatty Stock mice injected subcutaneously with 45 $\mu$g. DMBA at birth and painted once weekly with croton oil from the eighth week developed multiple skin papillomas. It is concluded that the DMBA acted as an initiator, since applications of croton oil following neonatal injections of aqueous gelatine induced no skin tumours. Applications of croton oil as well as DMBA injections were necessary for the development of skin papillomas: only one tumour was seen in the group injected with DMBA but painted with acetone. Thus, two factors were involved in skin carcinogenesis. Neonatally injected DMBA was the initiator and croton oil, applied to the skin, the promoter.

The initiating activity of 45 $\mu$g. DMBA injected into newborn mice was weaker than that of 150 $\mu$g. applied to the skin of adults. Tumour incidence was 53·3% in females and 40% in males treated when newly born: 91·3% in females and 82·4% in males treated at six weeks. The difference may be due to the dose of DMBA which actually reaches the skin being higher when it is applied directly to the skin of the adult. The interval before the appearance of the first papilloma was similar in both groups. Most skin tumours were benign, but one squamous cell carcinoma was seen in a mouse killed during the thirty-eighth week, which received one application to the skin of DMBA followed by thirty applications of croton oil. Malignant skin tumours, defined as those which had invaded the panniculus carnosus muscle, arose in long-term experiments in which mice were painted with DMBA and croton oil (Roe, 1956), and probably would have arisen in larger numbers in the present experiment if the animals had been observed for a longer period.

Mice given urethane (1 mg./g. body weight) when newly born followed by croton oil developed skin papillomas with an incidence significantly higher (44%) than mice given an equivalent dose of urethane subcutaneously at forty days and similar treatment with croton oil (19·5%). The mean latent period was practically the same. It was thought likely that different amounts of urethane reached the skin in mice treated at different times. Chieco-Bianchi and his colleagues (1963) believed that their results, which showed that groups receiving the same croton oil treatment had different tumour yields but almost the same latency, were consistent with the original hypothesis of Berenblum and Shubik (1947). On the basis of a two-stage theory of skin carcinogenesis, tumour incidence is a function of initiating action, while the latent period is a function of promoting action.

Skin tumour incidence in the "positive" control group of BALB/c mice, which received a single application of DMBA to the skin followed by twenty-five applications of croton oil, was very low. BALB/c mice therefore appear to be relatively insensitive to skin tumour induction by DMBA and croton oil: " 101 " strain mice painted with 150 $\mu$g. DMBA developed multiple papillomas after sixteen weeks of croton oil treatment (Roe and Peirce, 1961). The fact that few tumours arose in mice injected subcutaneously with DMBA when newly born, or as adults, may also be only an indication of the insensitivity of the strain, since a positive result was obtained using stock mice.

The mean number of lung tumours per mouse was significantly higher ($P = 0{\cdot}001$) in female stock mice injected at birth with DMBA and subsequently painted with croton oil, than in females which were treated with DMBA and acetone only. In male stock mice, however, and in BALB/c mice of both sexes, the difference was not significant. It is therefore doubtful whether croton oil can have a promoting effect on lung tumour induction.

Hepatomas, which occurred in a high proportion of male stock mice injected neonatally with DMBA, have rarely been induced by polycyclic hydrocarbons in adult animals. The induction of liver tumours by injecting polycyclic hydrocarbons into newborn mice has been discussed by Roe and Walters (1966).

The results reported here once again indicate that precautions should be taken in experiments in carcinogenesis to prevent the exposure of young animals to carcinogenic contaminants. Such contamination may both increase the incidence of so-called " spontaneous " tumours and initiate tumour formation in tissues such as the skin (Boutwell and Bosch, 1958; Roe, Bosch and Boutwell, 1958). If adventitious initiation occurs in this way tumour promoters may be mistaken for carcinogens.

### SUMMARY

Chester Beatty Stock mice of both sexes, injected at birth with 45 $\mu$g. DMBA in aqueous gelatine and treated from the age of six weeks with weekly applications to the skin of 0·1% croton oil in acetone for thirty weeks, developed multiple skin papillomas. The carcinogenic response was lower than in a group of mice treated with a single application to the skin of 150 $\mu$g. DMBA in acetone at six weeks followed by thirty applications of croton oil at weekly intervals. No skin papillomas were seen in mice injected neonatally with aqueous gelatine and subsequently painted with croton oil. Only one skin tumour was recorded in a group injected at birth with DMBA and treated with thirty weekly applications of acetone. It was concluded that neonatally-injected DMBA has initiating activity for the skin, but it is less potent than a direct application to the skin of a six-week-old mouse.

This investigation has been supported by grants to the Chester Beatty Research Institute (Institute of Cancer Research: Royal Cancer Hospital) from the Medical Research Council and the British Empire Cancer Campaign for Research, and by the Public Health Service Research Grant No. CA–03188–10 from the National Cancer Institute, U.S. Public Health Service.

### REFERENCES

Berenblum, I. and Shubik, P.—(1947) *Br. J. Cancer*, **1**, 383.
Boutwell, R. K. and Bosch, D.—(1958) *Cancer Res.*, **18**, 1171.
Chieco-Bianchi, L., Fiore-Donati, L., De Benedictis, G. and Tridente, G.—(1963) *Nature, Lond.*, **199**, 292.

GRAFFI, A., SCHARSACH, F. AND HEYER, E.—(1955) *Naturwissenschaften*, **42**, 184.
PIETRA, G., RAPPAPORT, H. AND SHUBIK, P.—(1961) *Cancer, N.Y.*, **14**, 308.
PIETRA, G., SPENCER, K. AND SHUBIK, P.—(1959) *Nature, Lond.*, **183**, 1689.
ROE, F. J. C.—(1956) *Br. J. Cancer*, **10**, 61.
ROE, F. J. C., BOSCH, D. AND BOUTWELL, R. K.—(1958) *Cancer Res.*, **18**, 1176.
ROE, F. J. C. AND PEIRCE, W. E. H.—(1961) *Cancer Res.*, **21**, 338.
ROE, F. J. C., ROWSON, K. E. K. AND SALAMAN, M. H.—(1961) *Br. J. Cancer*, **15**, 515.
ROE, F. J. C. AND WALTERS, M. A.—(1967) *Nature, Lond.*, **214**, 299.
TOTH, B., RAPPAPORT, H. AND SHUBIK, P.—(1962) *Proc. Soc. exp. Biol. Med.*, **110**, 881.—(1963) *J. natn. Cancer Inst.*, **30**, 723.
WALTERS, M. A.—(1966) *Br. J. Cancer*, **20**, 148.


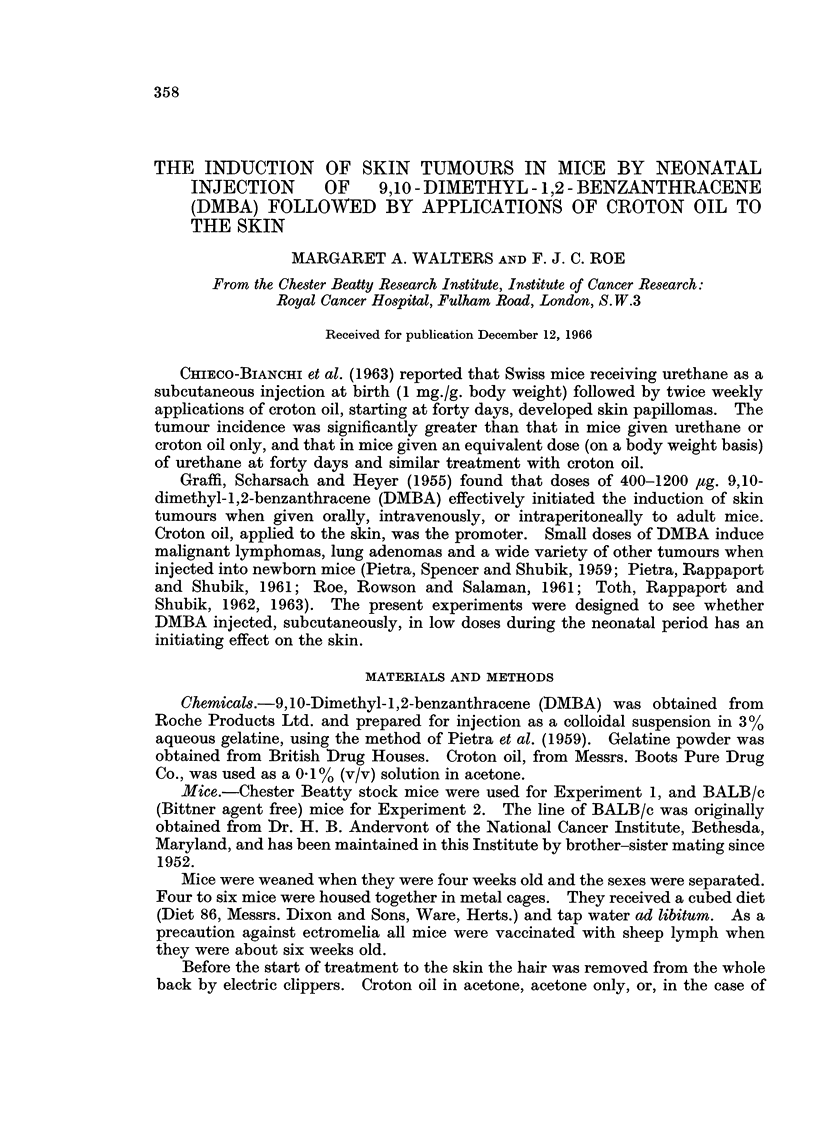

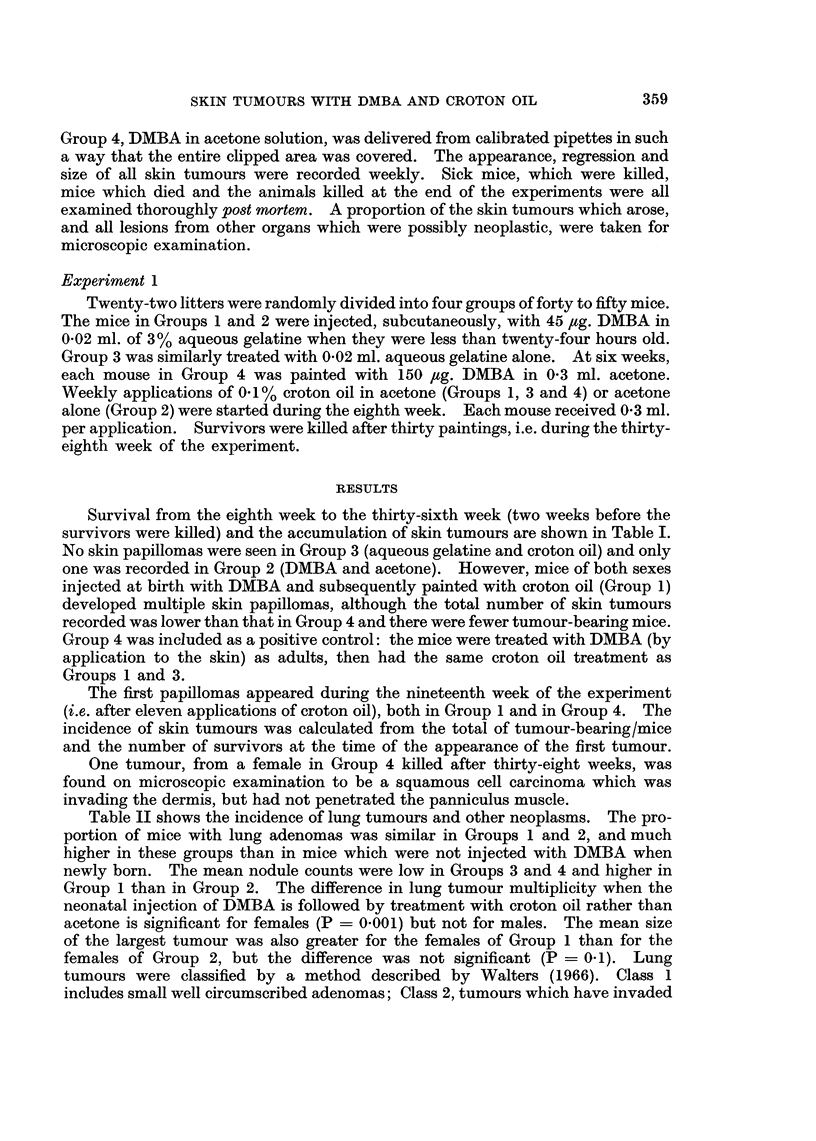

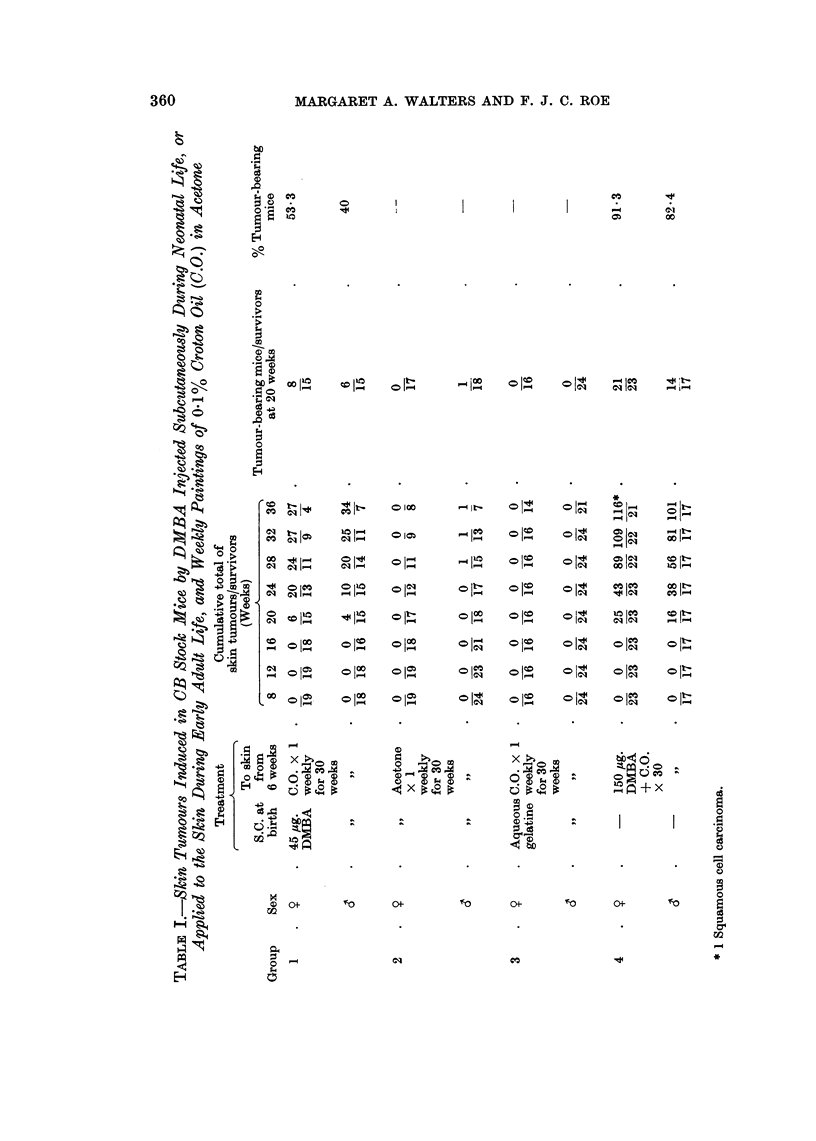

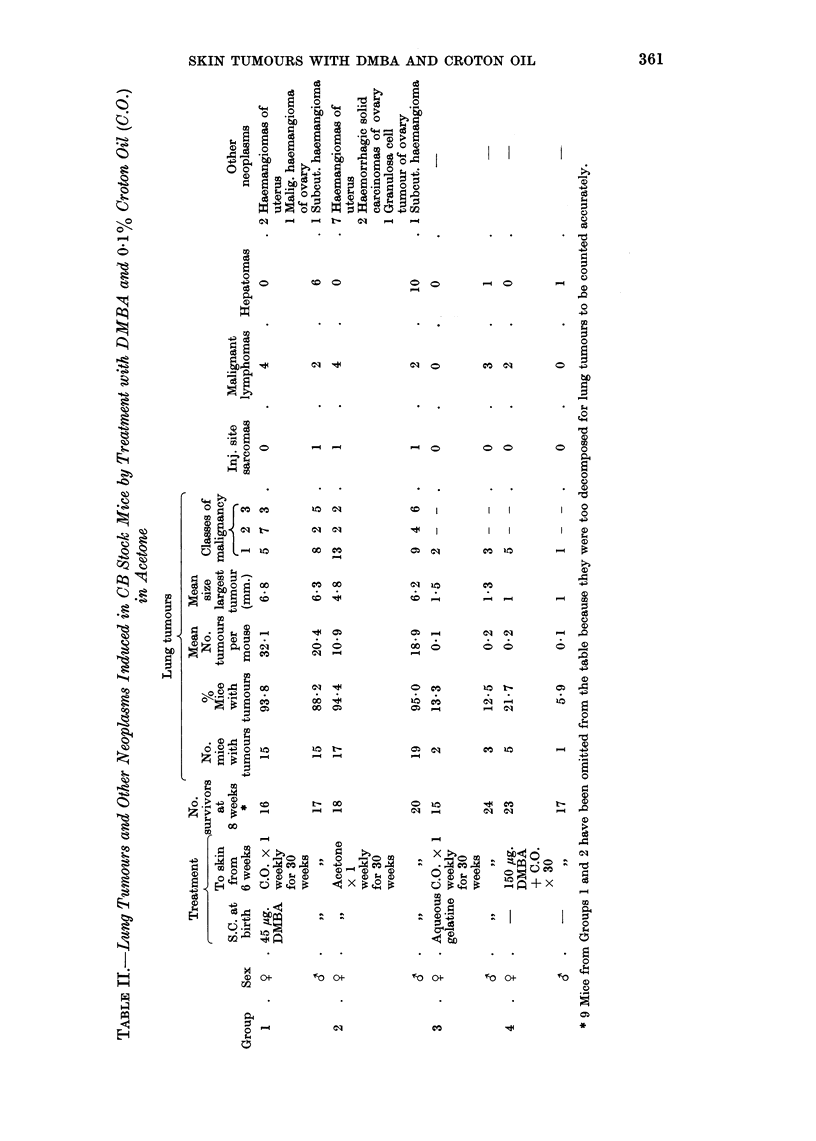

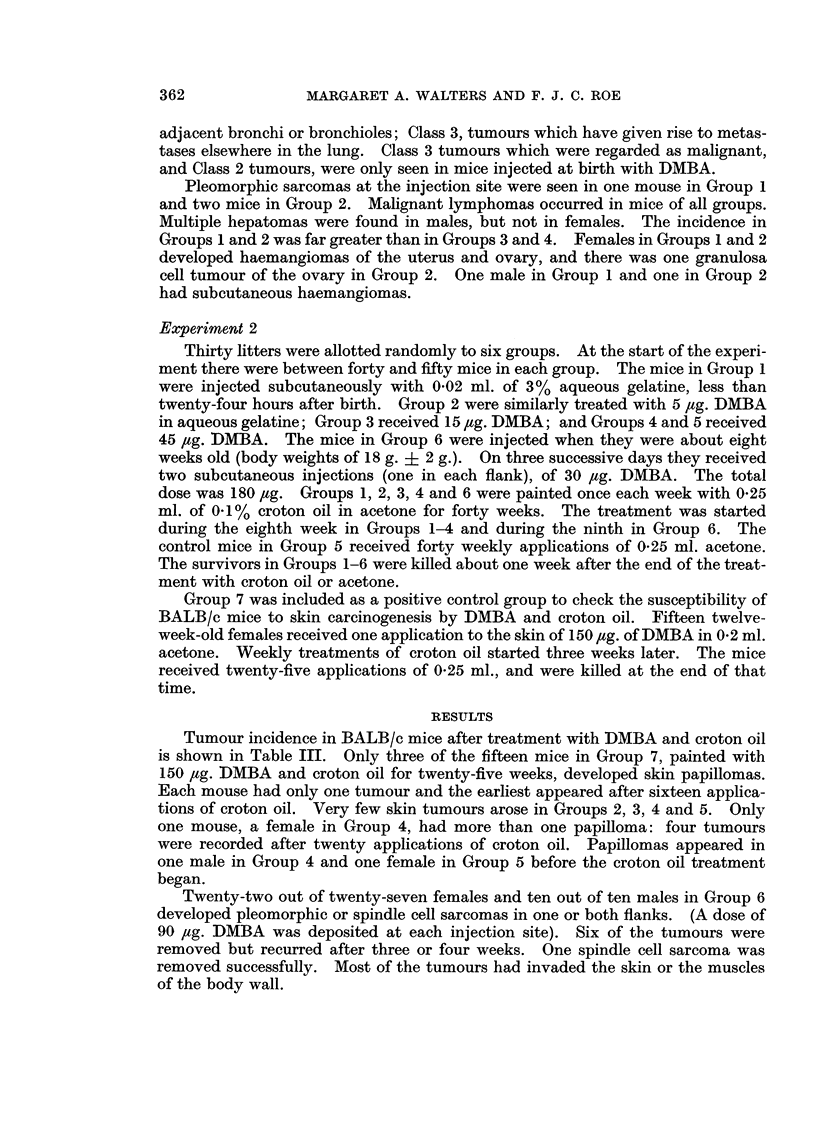

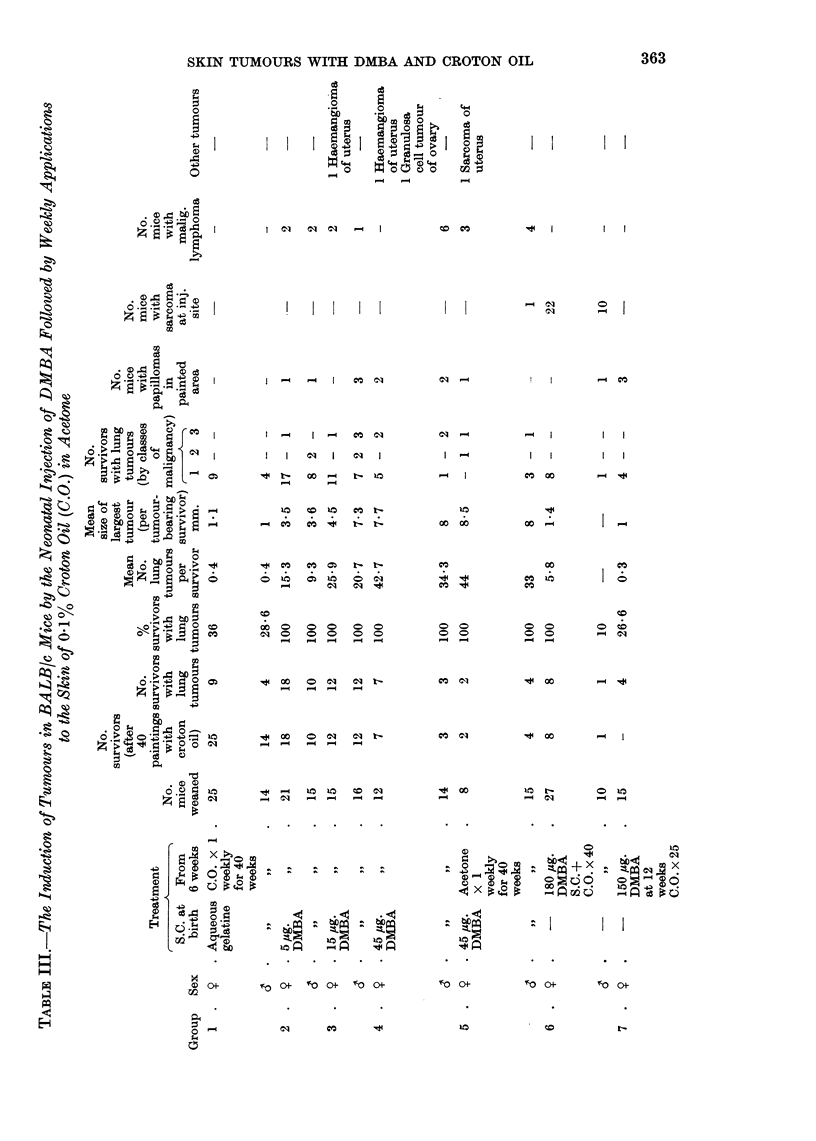

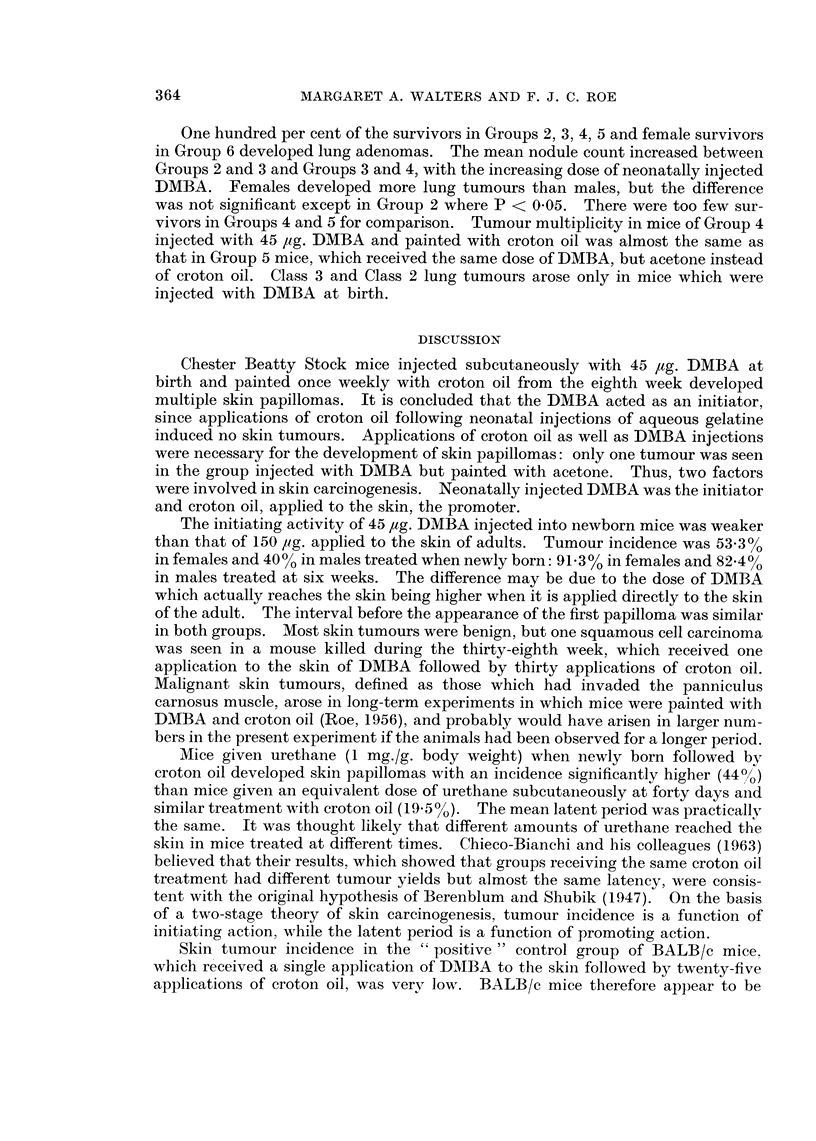

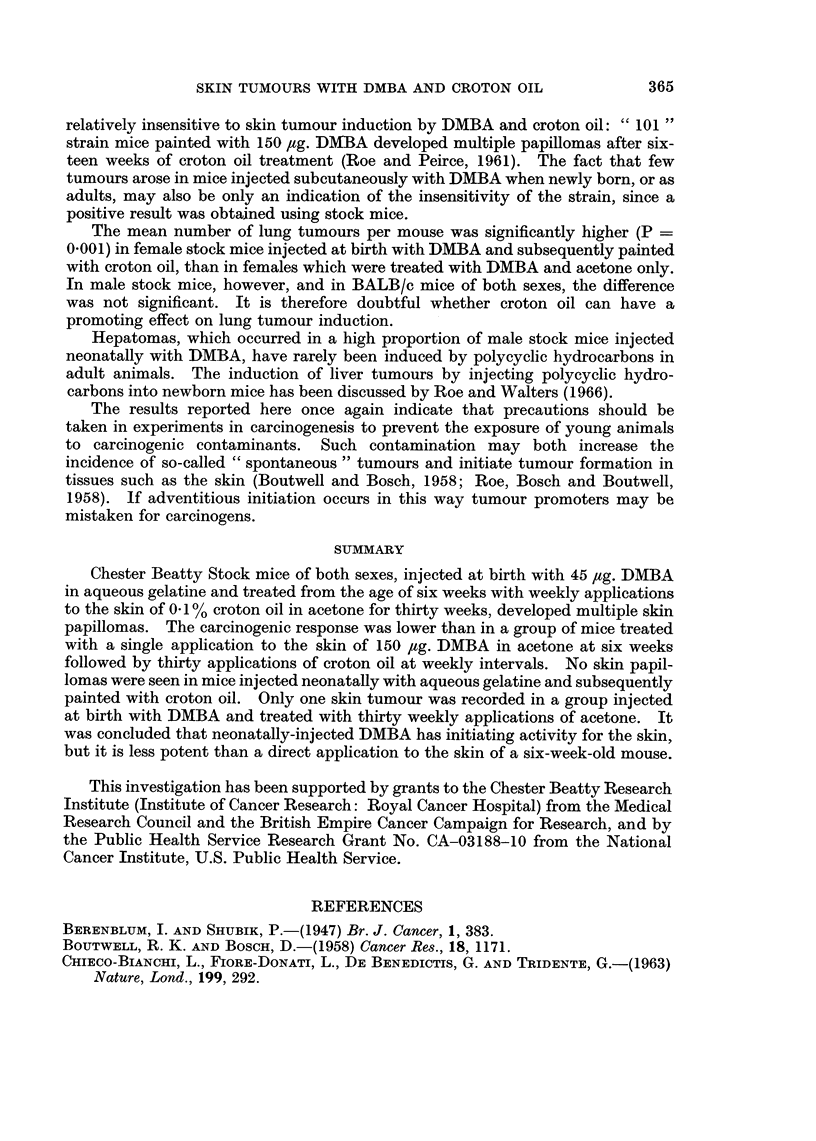

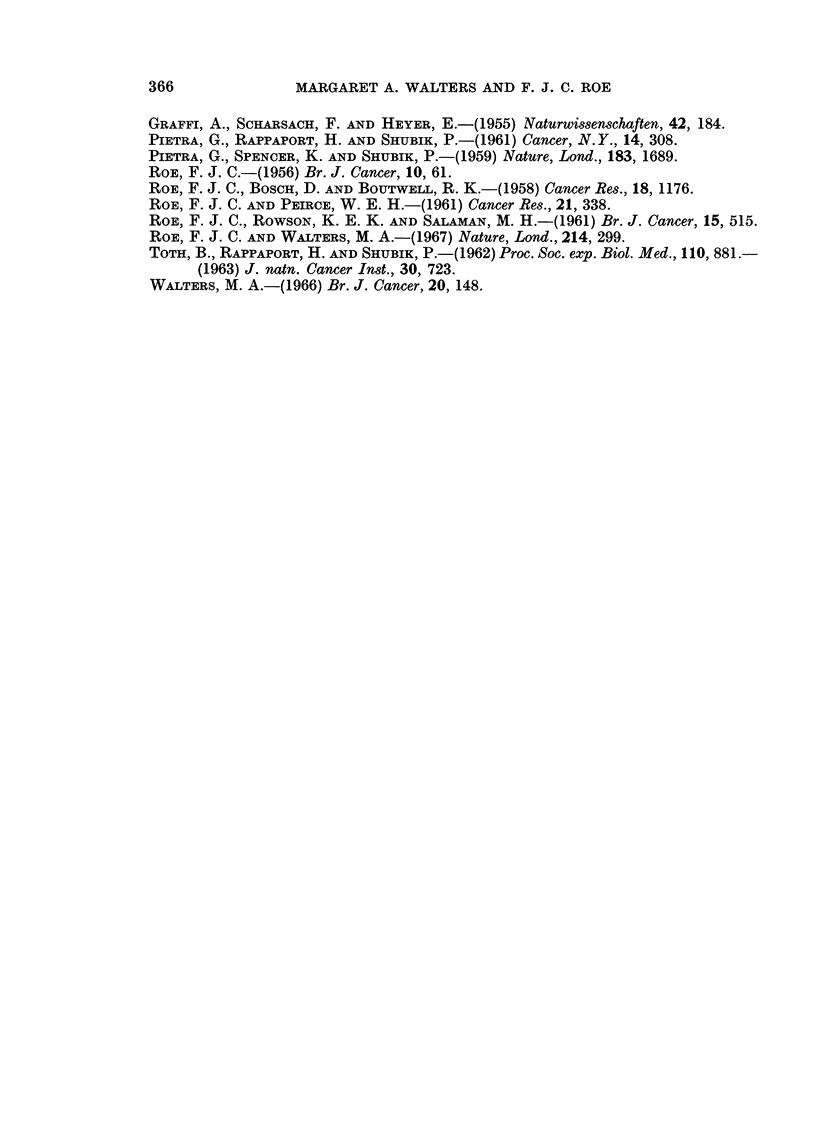


## References

[OCR_00887] BOUTWELL R. K., BOSCH D. K. (1958). The carcinogenicity of creosote oil: its role in the induction of skin tumors in mice.. Cancer Res.

[OCR_00891] CHIECO-BIANCHI L., FIORE-DONATI L., DEBENEDICTIS G., TRIDENTE G. (1963). INFLUENCE OF URETHANE ON SUSCEPTIBILITY TO LEUKEMIA INDUCTION BY GRAFFI VIRUS IN ADULT MICE.. Nature.

[OCR_00896] PIETRA G., RAPPAPORT H., SHUBIK P. (1961). The effects of carcinogenic chemicals in newborn mice.. Cancer.

[OCR_00897] PIETRA G., SPENCER K., SHUBIK P. (1959). Response of newly born mice to a chemical carcinogen.. Nature.

[OCR_00898] ROE F. J., BOSCH D., BOUTWELL R. K. (1958). The carcinogenicity of creosote oil: the induction of lung tumors in mice.. Cancer Res.

[OCR_00901] ROE F. J., PEIRCE W. E. (1961). Tumor promotion by Euphorbia latices.. Cancer Res.

[OCR_00903] ROE F. J., ROWSON K. E., SALAMAN M. H. (1961). Tumours of many sites induced by injection of chemical carcinogens into newborn mice, a sensitive test for carcinogenesis: the implications for certain immunological theories.. Br J Cancer.

[OCR_00906] TOTH B., RAPPAPORT H., SHUBIK P. (1962). Accelerated development of malignant lymphomas in AKR mice injected at birth with 7,12-dimethylbenz (a) anthracene.. Proc Soc Exp Biol Med.

[OCR_00910] Walters M. A. (1966). The induction of lung tumours by the injection of 9,10-dimethyl-1,2-benzanthracene (DMBA) into newborn suckling and young adult mice. A dose response study.. Br J Cancer.

